# Current patterns of care at adult emergency department in Ethiopian tertiary university hospital

**DOI:** 10.1186/s12245-023-00502-3

**Published:** 2023-04-11

**Authors:** Kibur Tarkie, Kassaye Demeke Altaye, Yophtahe Woldegerima Berhe

**Affiliations:** 1 Department of Internal Medicine, University of Gondar, Gondar, Ethiopia; 2 Department of Emergency Medicine and Critical Care, University of Gondar, Gondar, Ethiopia; 3 Department of Anesthesia, University of Gondar, Gondar, Ethiopia

**Keywords:** Emergency department, Care in emergency department, Emergency care

## Abstract

**Background:**

The complexity and demands of emergency healthcare service are continuously increasing, and it is important to regularly track the patterns of care at the emergency department (ED).

**Methodology:**

A retrospective study was conducted at the ED of the University of Gondar Comprehensive Specialized Hospital (UoGCSH) from April 1 to June 30, 2021. Ethical approval was obtained from the Emergency and Critical Care Directorate of UoGCSH. Data was collected from the emergency registry and descriptive analysis was performed.

**Results:**

A total of 5232 patients have visited and triaged at the ED. All patients who visited the ED have received triage service within 5 min of arrival. The average length of stay at the ED was 3 days. About 79.1% of patients have stayed at the ED beyond 24 h, and the unavailability of beds at admission areas was responsible for 62% of delays. Mortality rate at the ED was 1.4%, and male to female ratio of death was 1.2 to 1. Shock (all types combined), pneumonia with/without COVID-19, and poisoning were the leading causes of death at the ED which were responsible for 32.5%, 15.5%, and 12.7% of deaths respectively.

**Conclusions:**

Triage has been done within the recommended time after patient arrival. However, many patients were staying at the ED for an unacceptably prolonged time. Unavailability of beds at the admission areas, waiting long for senior clinicians’ decisions, delays in investigation results, and lack of medical equipment were the causes of delayed discharge from the ED. Shock, pneumonia, and poisoning were the leading causes of death. Healthcare administrators should address the lack of medical resources, and clinicians should provide timely clinical decision and investigation results.

## Introduction

The complexity and demands of emergency healthcare service are continuously increasing, and it is important to track the trends in the emergency department [1–3]. Emergency care delivery is an effective strategy to reduce the global burden of diseases. It could address 54 to 90% of deaths and 900 million to 2.5 billion disability-adjusted life years in low- and middle-income countries [4].

Emergency care is an often overlooked, but essential component of universal health coverage (UHC). Particularly for vulnerable populations, emergency care is often the last chance for the health system to save a life. According to human rights approach, access to the highest attainable emergency care is a human right [4]. About 90% of the burden of death and disability from injuries occurs in low- and middle-income countries [5]. The bulletin of World Health Organization (WHO) reported that vulnerable people who are unable to access health care will seek emergency care for acute conditions and exacerbations of chronic diseases which is the only available means of care for them [6]. The emergency department (ED) is a resource-intensive area; should be organized and equipped well to provide efficient and quality of emergency care [7, 8].

The global estimate of ED death is 15–16% of hospital mortality. Most of the ED deaths occur in low- and middle-income countries in which ED deaths occur over 5% higher rates compared to high-income countries [9, 10]. Previous studies in low-income settings have identified cardiovascular diseases, trauma, shock, respiratory diseases, and cancer as the leading causes of death in the ED [11–13].

The WHO urges all countries, regardless of resources and economic development, must begin by ensuring a comprehensive emergency care system and should continuously evaluate progress [4]. According to the Ethiopian Hospital Services Transformation Guideline, emergency patients should access the triage area without the hindrance of their financial capacity and/or security guard. Patients should obtain triage service by Emergency Triage Officer (nurse/physician) immediately after arrival. Patients that are not classified as emergency cases should be referred to the Central Triage. An ED must have 2–3 resuscitation couches with 1:1 nurse to patient ratio. After resuscitation or patients who require temporary observation and management will be admitted to the Emergency patient Observation/Treatment area with 5–10 beds as a minimum. Patients kept in this area need frequent evaluation by the ED physician, available senior clinicians, and nurses. The recommended nurse to patient ratio is 1:3. Patients enter the emergency case management pathway upon disposal from the Emergency Triage Officer. Appropriate care is then initiated by the emergency case management team. Patients should stay at the ED for the maximum of 24 h. However, due to different reasons, those emergency service standards might not be met [14]. The general objective of this study was to evaluate the current patterns of care at adult emergency department in the University of Gondar Comprehensive Specialized Hospital (UoGCSH), Ethiopia, 2021.

## Methodology

A descriptive retrospective study was conducted at the emergency department of the University of Gondar Comprehensive Specialized Hospital (UoGCSH) from April 1 to June 30, 2021. The UoGCSH is found in Gondar town and the most senior tertiary hospital in the Northwest Ethiopia. The hospital is only a tertiary hospital serving 3 zones (North Gondar, Central Gondar, and West Gondar) that have a population of over 5 million people in cumulative. The emergency department was organized in “Triage, Medical Wing, and Surgical Wing.” The triage was 3-level system and further partitioned into “Red Zone, Orange Zone, and Yellow Zone” in order to facilitate care delivery according to severity and criticality of patients’ conditions and appropriate utilization of medical resources.

The triage was organized with 2 examination couches and 3 nurses at a time. The red, orange, and yellow zones were organized with 10, 30, and 15 beds and 3, 2, and 3 nurses respectively at a time. A total of 24 physicians (medical, surgical, and general practitioners), 25 interns, 4 laboratory technologists, 4 pharmacists, and 28 non-clinical staff (janitors and porters) were actively deployed at the ED in rotation. The available laboratory investigations at the ED’s laboratory were hematocrit, blood film, blood group with cross-match, random blood sugar, urinalysis, stool examination, and pregnancy test. Other tests were investigated at the central laboratory. All data of patients who visited the ED were included in this study. The variables that we collected data for were triaging time, triage level, diagnosis, length of stay at the ED, ED mortality, causes of death, and causes for delayed discharge from the ED. Data which were incomplete for those targeted variables were excluded from the study. We did not collect data for variables such as chief complaints, and time points (collection of investigation samples/imaging, time of decision to admission and actual admission, time of arrival (day/night), and time to collect investigation results). We aimed to collect data of those variables but they were unavailable in the registry.

All consecutive patients who had visited the adult ED at the UoGCSH during the study period were included in this study. Ethical approval was obtained from the Emergency and Critical Care Directorate, College of Medicine and Health Sciences, University of Gondar (reference number: ECCD/021/03/2021). Confidentiality was ensured by removing identifiers. All methods were performed in accordance with the relevant guidelines and regulations.

Data collection was executed by 2 medical residents from a manual emergency registry of the hospital under supervision of the investigators. The collected data was checked, cleaned, and analyzed by using SPSS version 20 software (IBM Corporation). Descriptive analysis was performed and present results in tables and graphs.

## Results

A total of 5232 patients have visited and triaged at the ED at UoGCSH from March 1 to May 31, 2021. Of the total, 3196 (61.1%) were medical patients who were evaluated at one of the red (463 (14.5%)), orange (1052 (32.9%)), or yellow (1681 (52.6%)) zones of the medical wing. The remaining 2036 (39.9%) patients were identified as surgical and transferred to the surgical wing. Thirty-eight hundred and nineteen (73%) patients were aged between 15 and 64 years old, and the remaining 27% were 65 years old and above. The majority of patients (56.4%) were males.

**Fig. 1:**
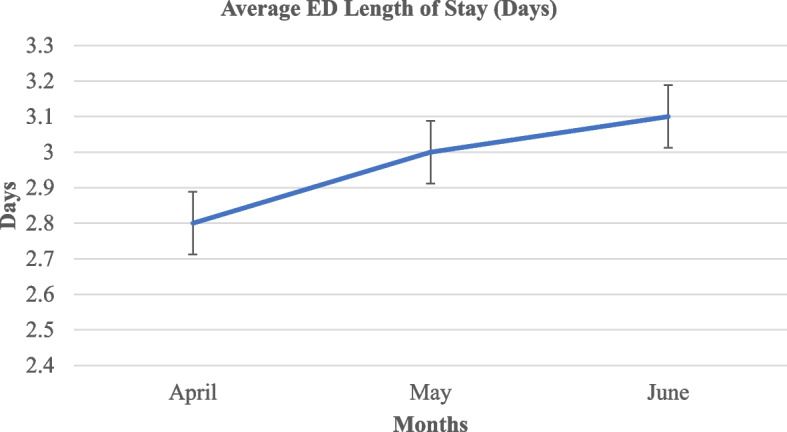
A line graph showing the average length of stay at the emergency department in days

All patients who visited the ED have received triage service within 5 min of arrival. The average length of stay at the ED was three days (Fig. 1). Among the medical patients, only 670 (20.9%) were discharged from the ED to home within 24 h, and 2526 (79.1%) stayed at the ED beyond 24 h. After staying for over 24 h, the largest proportion of patients (2286 (90.5%)) were discharged from the ED to home (Table 1). Our data showed that unavailability of free beds in the admission areas was the commonest cause for longer stay at the ED beyond 24 h. It was responsible for 62% of delays to discharging from ED. About 15% of delays were caused by waiting for senior clinicians’ decisions (Table 2).

**Table 1:** Patterns of discharging from the ED, UoGCSH, *N* = 3196

Discharging from the ED	Frequency (%)
Patients discharged from the ED to home within 24 h	670 (20.9)
Patients stayed at the ED for > 24 h	2526 (79.1)
Patients’ destination after discharging from the ED (*n* = 2529)	
Home	2286 (90.5)
Wards	196 (7.8)
Intensive care units	44 (1.7)

**Table 2:** Causes of delayed discharging from the ED, UoGCSH, *N* = 2526

Causes	Frequency (%)
Unavailability of free beds in admission areas	1566 (62)
Waiting for senior clinicians’ decision	379 (15)
Delayed investigations and tests	328 (13)
Lack of equipment (oxygen, monitoring, suction machines, and other ancillary medical equipment)	253 (10)

**Fig. 2:**
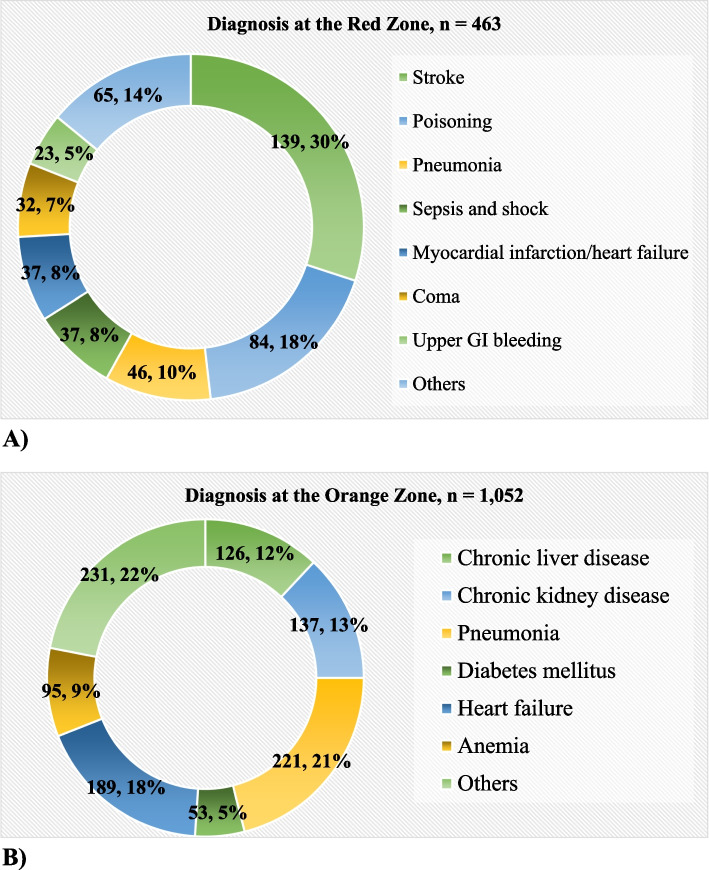
Pie charts showing the commonest diagnosis at the emergency department. **A** At the red zone. **B** At the orange zone

Stroke was the commonest diagnosis at the red zone followed by poisoning and pneumonia. In the orange zone, pneumonia was the commonest diagnosis while heart failure and chronic kidney disease were the second and third common diagnoses (Fig. 2). Testing for HIV/AIDS was performed upon physician recommendation for patients who were identified as high risk according to the Ethiopian National Guideline for HIV/AIDS Prevention and Treatment. A total of 266 tests were done, and out of the tested patients, 13 (4.9%) were found to have positive status. The ED had received 258 road traffic accident victims; out of which 238 (92.2%) were vehicle occupants, 20 (7.8%) were motor cyclists, and no pedestrian.

A total of 71 (1.4%) deaths occurred at the ED. Male to female ratio among ED deaths was 1.2 to 1. Pneumonia with/without COVID-19 was the commonest cause of death at the ED which accounts for 11 (15.5%) of deaths. However, shock (all types combined) was responsible for the majority of ED death 23 (32.5%). Following pneumonia, poisoning (9 (12.7%)), cardiogenic shock (9 (12.7%)), septic shock (7 (9.9%)), and hypovolemic shock (7 (9.9%)) were among the top five leading causes of death at the ED (Table 3).

**Table 3:** Causes of death at the ED, UoGCSH, 2021, *N* = 71

Causes of death	April, *n* (%)	May, *n* (%)	June, *n* (%)	Total *n* (%)
Pneumonia + / − COVID-19	5 (19.2)	4 (17.4)	2 (9.1)	11 (15.5)
Bullet/stab injury	0	1 (4.3)	1 (4.5)	2 (2.8)
Cardiogenic shock	1 (3.8)	2 (8.7)	6 (27.3)	9 (12.7)
Septic shock	2 (7.7)	2 (8.7)	3 (13.6)	7 (9.9)
Hypovolemic shock	4 (15.3)	2 (8.7)	1 (4.5)	7 (9.9)
ARDS	2 (7.7)	0	0	2 (2.8)
HIV/AIDS	1 (3.8)	1 (4.3)	0	2 (2.8)
Diabetes mellitus	2 (7.7)	1 (4.3)	1 (4.5)	4 (5.6)
Stroke	2 (7.7)	2 (8.7)	1 (4.5)	5 (7.0)
Poisoning	2 (7.7)	4 (17.4)	3 (13.6)	9 (12.7)
Anemia	1 (3.8)	0	0	1 (1.4)
Cerebral malaria	0	0	1 (4.5)	1 (1.4)
Gastric cancer	1 (3.8)	0	1 (4.5)	2 (2.8)
Brain cancer	0	1 (4.3)	0	1 (1.4)
Meningitis	1 (3.8)	1 (4.3)	1 (4.5)	3 (4.2)
Head injury	1 (3.8)	1 (4.3)	0	2 (2.8)
Viral hepatitis	1 (3.8)	0	0	1 (1.4)
Acute renal failure	0	0	1 (4.5)	1 (1.4)
Hypoglycemia	0	1 (4.3)	0	1 (1.4)
**Total ** **(% within months)**	**26 (100)** **(36.6)**	**23 (100)** **(32.4)**	**22 (100)** **(31.0)**	**71 (100)** **(100)**

## Discussion

Healthcare administrators and clinicians need to understand the trends in the ED as it allows for better resource planning [3]. Our study revealed that a larger proportion of patients who have visited the ED had medical conditions and the remaining had surgical conditions. All patients have been evaluated by the triage nurse within 5 min. This was a better achievement compared to that of French emergency national survey in which 50% have been seen by the triage nurse in less than 5 min [8]. The French survey was conducted among 734 EDs across France nationally on a very large population, whereas the current study was conducted in a single tertiary hospital.

The Ethiopian Hospital Services Transformation Guideline recommends to discharge patients out of the ED within 24 h. However, we found that a greater proportion of patients (79.1%) have stayed at the ED for a longer time. This finding is very high compared to a previous study that reported prolonged ED stay in 13.5% only. The previous study was done in multiple trauma centers in the USA and considered ED stay as prolonged if longer than 6 h [15]. Another study has also reported that 42.3% of patients had prolonged ED stay. This could be due to the presence of another governmental tertiary hospital in the catchment area that could share the burden of emergency diseases; as a result, there was lesser patient flow [16]. Additionally, there was recent armed conflicts in the nearby region had substantially increased the patient loads at the ED. Patients were staying at the ED for an average of 3 days and we found it considerably longer compared to other EDs with better facility which reported average length of stay at the ED was 167 min [15], 198 min [17], 229 min [18], 247 min [19], and 344 min [20].

Prolonged ED stay is associated with poor quality of medical care, patient dissatisfaction, higher rates of adverse events, and mortality [21]. We found that unavailability of beds in admission areas, delayed senior clinicians’ decision and investigation results were the commonest reasons for prolonged stay at the ED. Multiple studies reported that unavailability of beds in admission areas and delayed laboratory and imaging investigations were the leading causes of prolonged stay at the ED [16, 19, 22, 23]. Clinicians in Netherlands and Pakistan have perceived that increased patients’ complexity, long treatment times, and poor availability of inpatient beds were the leading causes of crowding in the ED [24]. A study in Beijing has identified persistent expectations of receiving ongoing care in the ED for minor illnesses and access block as a result of inability to move patients for inpatient care due to lack of bed [25]. Therefore, effective collaboration between the ED and downstream wards may reduce prolonged stay at the ED [26].

The commonest diagnosis in the red zone was stroke, poisoning, and pneumonia while pneumonia, heart failure, and chronic kidney disease at the orange zone. In a study done in the USA, abdominal issues, mental health issues, and respiratory tract infections were found to be the top reasons for visiting the ED [3]. A study done in Tanzania revealed that respiratory tract infections, urinary tract infections, and undifferentiated febrile illnesses were the commonest diagnoses at the ED [27]. A study done in Denmark reported injury, poisoning, symptom and signs, circulatory diseases, and respiratory diseases were the top five frequent diagnoses at the ED [28]. Despite the similarities, the inconsistencies might be explained by the differences in study populations, socio-economic, and geographical determinants of health.

Seventy-one (1.4%) deaths occurred at the ED during the study period. This mortality rate is comparable to previously reported rates in low- and middle-income countries [10, 13]. However, higher mortality rates were also reported by previous studies which counted death on arrival as an ED death [11, 12]. In our study, we included only patients who had signs of life during arrival at the ED and died later. Hospital setup differences also might be the possible reasons for the discrepancies. Studies conducted in the sub-Saharan Africa reported a higher average mortality rate (3.4%) as they included pediatric emergency mortality [10]. Higher ED mortality (4.5%) was reported among critically injured patients [15], and 21.8% among older patients [29]. However, the mortality rate in our ED was higher compared to those found in high-income countries 0.1% in France [8]. We also found that males had relatively higher mortality rate than females at the ED (ratio = 1.2:1) and it is supported by multiple previous studies (1.2:1 [11]; 1.4:1 [13]; 1.3:1 [10]; 1.3:1 [12]). This could be due to higher exposure of males to situations that might lead to emergency conditions such as trauma [13].

Pneumonia with/without COVID-19 was the leading cause of death (15.5%) followed by poisoning and different types of shock. Respiratory diseases are regularly mentioned as one of the commonest causes of ED death, predominantly during the current ongoing COVID-19 pandemic era [11–13, 15, 28, 29]. We even expect more undetected pneumonia with COVID-19 mortality as our resource-limited setup in a low-income country had very low screening capacity. In cumulative, shock (all types combined) has killed the largest proportion of patients who died at the ED (32.5%). Among the types of shock, cardiogenic shock was the most common cause of death at the ED followed by septic shock and hypovolemic shock. Shock is commonly found in the top lists of causes of death at EDs [11–13, 15, 30].

Another commonest cause of death at our ED was poisoning. Nine (12.7%) patients have died at the ED due to poisoning (male:female = 1:2). Eight of them (88.9%) were aged between 15 and 29 years old and only 1 (11.1%) patient was aged over 30 years. Furthermore, all patients who died due to poisoning were committing suicide. Studies have shown that mental health issues and poisoning are common causes of visit and death at the ED [3, 11, 28], and most patients poisoned themselves intentionally [31]. Suicidal ideation and attempts are highly prevalent in Ethiopia and more common among individuals with mental health issues [32, 33].

The limitations of this study are missing some variables such as chief complaints due to lack of data during retrospective collection and no inferential statistics to determine factors associated with outcome variables. It was also a single-center study in a short period of time.

## Conclusions

Patients who were visiting the ED were finding triage services within the recommended time. However, large proportion of patients were staying at the ED for unacceptably prolonged time irrespective of the national guideline recommendations. Unavailability of beds at the wards, delays in investigation results, waiting long for senior clinicians’ decisions, and lack of medical equipment were the commonest causes of delayed discharge from the ED. Shock (all types combined), pneumonia with/without COVID-19, and poisoning were the leading causes of death at the ED. Healthcare administrators should address the lack of medical resources, and clinicians should act to provide timely clinical decision and arrival of investigation results.

## Data Availability

Data and materials used in this study are available and can be presented by the corresponding author upon reasonable request.

## Abbreviations

ARDS: Acute respiratory distress syndrome
COVID-19: Coronavirus disease-2019
ED: Emergency department
SPSS: Statistical Package for the Social Sciences
UHC: Universal Health Coverage
UoGCSH: University of Gondar Comprehensive Specialized Hospital
WHO: World Health Organization
